# AhIRT1 and AhNRAMP1 metal transporter expression correlates with Cd uptake in peanuts under iron deficiency

**DOI:** 10.1371/journal.pone.0185144

**Published:** 2017-10-05

**Authors:** Chu Chen, Shenglan Xia, Rubo Deng, Caifeng Liu, Gangrong Shi

**Affiliations:** College of Life Sciences, Huaibei Normal University, Huaibei, P. R., China; National Botanical Research Institute CSIR, INDIA

## Abstract

Fe deficiency may increase Cd accumulation in peanuts. However, the mechanisms are not yet fully understood. In the present study, two contrasting peanut cultivars, Luhua 8 (low seed-Cd cultivar) and Zhenghong 3 (high seed-Cd cultivar) were used to investigate the effect of Fe deficiency on the uptake and accumulation of cadmium (Cd) by hydroponic experiments. Under Fe-sufficient conditions, compared with Luhua 8, Zhenghong 3 had higher specific root length (SRL) and proportion of fine roots with a lower *K*_m_ for Cd and showed slightly higher expression of *AhIRT1* and *AhNRAMP1* in the roots. These traits may be responsible for high capacity for Cd accumulation in Zhenghong 3. Under Fe deficiency, the increase of Cd accumulation was much larger in Zhenghong 3 than in Luhua 8. Kinetics studies revealed that the *V*_max_ for Cd influx was 1.56-fold higher in Fe-deficient plants than in Fe-sufficient plants for Zhenghong 3, versus 0.48-fold higher for Luhua 8. Moreover, the increased expression levels of *AhIRT1 *and *AhNRAMP1* induced by Fe deficiency was higher in Zhenghong 3 than in Luhua 8. Yeast complementation assays suggested that the AhIRT1 and AhNRAMP1 may function as transporters involved in Cd uptake. In conclusion, the different Cd accumulation between the two cultivars under Fe deficiency may be correlated with *V*_max_ value for Cd uptake and the expression levels of *AhIRT1* and *AhNRAMP1* in the roots.

## Introduction

Iron (Fe) is an important microelement for plant growth and development. As a redox-active metal, Fe is involved in many physiological processes including photosynthesis, mitochondrial respiration, nitrogen assimilation, hormone biosynthesis, production and scavenging of reactive oxygen species, osmoprotection and pathogen defence [1, 2]. Although the total Fe content in soil regularly exceeds plant requirements, it is present as oxihydrates with low bioavailability [3], particularly in calcareous soils, which represent 30% of the earth's surface [4]. Fe deficiency has become a yield-limiting factor for a variety of field crops all around the world.

Cadmium (Cd) is a highly toxic non-essential metal that is easily taken up by plant roots and transported into the aerial parts [5]. In the root, Cd is taken up by epidermal cells, radially transferred to the inner parts of the root via both apoplastic and symplastic pathway. Cd is loaded from the symplasm into the xylem by Fe transporters such as the iron-regulated transporter [6–8] and natural resistance-associated macrophage protein (NRAMP) [9–11]. Fe deficiency has been demonstrated to induce a high expression of genes of IRT and NRAMP in plant roots, leading to a considerable increase in the uptake and accumulation of Cd [7, 8, 12]. In peanuts, *AhIRT1* and *AhNRAMP1 *have been identified as Fe transporters [13, 14]. The expression level of *AhIRT1* and *AhNRAMP1* were obviously induced by iron deficiency in the roots. Yeast complementation assays suggested that *AhNRAMP1* and *AhIRT1* encode functional iron transporter. The tobacco transgenic lines with the induced expression of *AhNRAMP1* showed enhanced tolerance to iron deprivation.

Plant roots show a particularly high morphological plasticity in response to Fe deficiency. In the reference plant Arabidopsis (*Arabidopsis thaliana*), a mild deficiency of Fe increased root elongation; however, severe Fe deficiency caused stunting of roots [15]. Fe deficiency can enhance the formation of root hairs [2, 16], increase root diameter [16], and promote development of lateral roots [2]. Additionally, several studies have illustrated that root morphological characteristics, such as the root lengths, surface areas (SA), specific root lengths (SRL) and number of root tips, and root diameters (RD), significantly relate to the uptake and accumulation of Cd in plants [5, 17–19].

Peanut (*Arachis hypogaea* L.) is one of the most important oilseed and food crops worldwide. It is grown on nearly 24 million hectares of land areas globally with an annual production of 38 million tons [20]. Extensive studies have shown that peanut has particularly high capacity for accumulating Cd in both the seed and vegetative tissues, and the ability of Cd accumulation varies among cultivars [5, 21–25]. It was also demonstrated that Fe deficiency dramatically increased Cd accumulation in plant tissues of peanuts [25–27]. The accumulation of Cd in peanuts was associated with the root morphological characteristics [5, 19]. However, the mechanisms involved in Fe deficiency-induced increase of Cd accumulation in peanuts are not yet fully understood. Based on the abovementioned results, we hypothesized that Fe deficiency may induce higher expression of *IRT* and *NRAMP* and changes of root morphology in peanut as previously reported in other plant species, resulting in an increase of Cd uptake and accumulation in plants. However, to the best of our knowledge, this hypothesis has never been tested experimentally.

The present study aimed to (i) characterize the physiological aspects of Cd uptake in the two most contrasting peanut cultivars identified in our previous work; (ii) evaluate the effects of Fe deficiency on the kinetics of Cd influx, root morphology and the expression of *AhIRT1* and *AhNRAMP1* in the roots of peanut; and (iii) discriminate the contributions of root morphology and of Fe transporters to the increased Cd uptake induced by Fe deficiency.

## Materials and methods

### Plant culture

Based on previous studies [28], two peanut cultivars differing in seed Cd accumulation, Luhua 8 (low seed-Cd cultivar) and Zhenghong 3 (high seed-Cd cultivar), were selected for this study. Seeds were sterilized with 1% sodium hypochlorite for 10 min, and then they were rinsed with tap water for 24 h and germinated on well-washed sand. After 5 days, the uniform sized seedlings were selected and transferred to the nutrient solution (pH 5.8) [5]. The nutrient solution was renewed every two weeks. Plants were cultivated in a chamber at a 14-h photoperiod (average irradiance of 600 μmol m^-2^ s^-1^), with day/night temperatures 25/20°C, and a relative humidity between 50% and 60%. The pots were randomly arranged daily during the growing period.

### Influence of Fe deficiency on plant growth and Cd accumulation

Seedlings were grown for 12 d in basal nutrient solution with (+Fe) or without (–Fe) 50 μM FeEDTA. Each treatment was replicated three times (pots) for each cultivar and the experiment was repeated three times. All treatments contained 0.2 μM CdCl_2_. The harvested seedlings were divided into roots and shoots. Roots were immersed in 20 mM Na_2_-EDTA for 15 min to remove metal ions adhering to the root surfaces. All plant parts were oven-dried for 30 min at 105°C, and then dried to a constant weight at 70°C. The concentrations of Cd in the dried samples were determined by flame atomic absorbance spectrometry (AAS) after digested in mixed acid [HNO_3_ + HClO_4_ (3:1, v/v)].

The translocation factors (*TF*s) of Cd from root to shoot and total Cd in the whole plant were calculated as follows:

$$TF={\left[\mathrm{Cd}\right]}_{\mathrm{shoot}}/{\left[\mathrm{Cd}\right]}_{\mathrm{root}}$$ (1)

$$\mathrm{Total}\;\mathrm{Cd}\;\mathrm{in}\;\mathrm{plants}\;={\left[\mathrm{Cd}\right]}_{\mathrm{shoot}}\times \mathrm{shoot}\;\mathrm{biomass}+{\left[\mathrm{Cd}\right]}_{\mathrm{root}}\times \mathrm{root}\;\mathrm{biomass}$$ (2)

### Influence of Fe deficiency on Cd uptake kinetics

Five-d-old seedlings with uniform sizes were transferred to 250 ml plastic pots (one seedling per pot). Seedlings were grown in full nutrient solution for 2 d. After this period, Fe deficiency was induced in one-half of the plants replacing the full nutrient solution with a nutrient solution without Fe for 12 d. The nutrient solution was then replaced with a pretreatment solution containing 2 mM MES (pH adjusted to 6.0 with KOH) and 0.5 mM CaCl_2_. After 24 h pretreatment, seedlings were exposed to ten concentrations of CdCl_2_ (0.2–60 μM) respectively. The uptake solutions also contained 0.5 mM CaCl_2_ and 2 mM MES (pH 6.0). Each treatment concentration was replicated three times. After 20 min uptake, the seedlings were quickly rinsed with the pretreatment solution, and then transferred to pots containing 100 ml of ice-cold desorption solution (2 mM MES, and 5 mM CaCl_2_) for 30 min. After desorption, seedlings were separated into roots and shoots. All plant samples were oven-dried for 30 min at 105°C, and then dried to a constant weight at 70°C. The dried root tissues were weighed and digested with mixed acid [HNO_3_ + HClO_4_ (3:1, v/v)]. Cd was determined by flame-atomic absorption spectrometry (AAS).

### Influence of Fe deficiency on leaf chlorophyll, active Fe content, and root morphology

Seedlings were grown in basal nutrient solution with (+Fe) or without (–Fe) 50 μM FeEDTA for 12 d. Each cultivar and treatment was replicated three times (pots). Mature leaves (0.2 g) from two plants in each pot were extracted in the dark at 4°C in a 5-ml mixture of acetone and ethanol (v/v = 1:1) until the color had disappeared. Light absorbance at 663 and 645 nm was determined by spectrophotometry. Chlorophyll contents (Chl_*a+b*_) as the sum of chlorophyll a and b contents were calculated according to Lichtenthaler [29].

Active Fe content was determined according to the procedure of Takker and Kaur [30]. Fresh young leaves were cut into pieces and extracted with 1 M HCl (in 1:10 tissue: extractant), shaken for 5 h and filtered, and the Fe concentration in the filtrate was measured with AAS.

Detopped root systems were scanned by using a root automatism scanning apparatus (MIN Mac, STD1600+) as the method described by Lu et al. [5]. The root lengths [17], surface area (SA), root diameters (RD), and root volumes (RV) were measured from the root images using the WinRHIZO^TM^2000 software (Regent Instruments, QC, Canada). Specific root length (SRL, m g^-1^) was calculated as the ratio of RL to root dry biomass. Five root diameter classes with an interval width of 0.2 mm were defined to determine the root hierarchical architecture. According to Lu et al. [5], the roots with diameter less than 0.4 mm were defined as the fine roots, and their proportions in root system were calculated on the basis of RL in different diameter classes.

### Real-time quantitative PCR

Seedlings were grown in basal nutrient solution with (+Fe) or without (–Fe) 50 μM FeEDTA for 12 d. Each cultivar and treatment was replicated three times (pots). Total RNA was extracted from the roots of Fe-deficient or Fe-sufficient seedlings by using TRIzol reagent (Takara, Japan). First-strand cDNA was synthesized from 1 μg of total RNA, using PrimeScript RT reagent kit with gDNA Eraser (Perfect Real Time) (Takara, Japan). Quantitative real time PCR (qRT-PCR) was performed on ABI 7300 system (Applied Biosystems, USA), using the SYBR Premix Ex Taq kit (Takara, Japan) according to the manufacturer’s protocol. The primers used for qRT-PCR are listed in Table 1. The peanut *Actin* gene was used as an internal control for normalization of gene expression. Each experiment was replicated three times.

**Table 1:** The primers used for qRT-PCR and yeast analysis in this study.

Primer name	Directions	Sequence (5’–3’)
Ahactin-qPCR	Forward	CTGAAAGATTCCGATGCCCTGA
	Reverse	AACCACCACTCAAGACAATGTTACCA
AhNRAMP1-qPCR	Forward	TTACTCCCAAACTCAGTGGTCAAG
	Reverse	GTGGAGGAAGAGGTTGTGCG
AhIRT1-qPCR	Forward	GTTCTCTGCCTTATTCACGCTCAT
	Reverse	GCCAACACTAACAACAACACCCAT
AhNRAMP1-CDS	Forward	GGGGTACCATGGCAAGCGTTCTTAGACAGC
	Reverse	CCGCTCGAGTTATTCCGGTAGTGGGATATCAGC
AhIRT1-CDS	Forward	CGAGCTCATGGGTACTAATTCAGAAGTAAAAC
	Reverse	GCTCTAGATTAATTCCATTTTGCCATGA

### Functional analysis of AhIRT1 and AhNRAMP1 in yeast

The full-length coding regions of *AhIRT1* and *AhNRAMP1* were amplified by PCR with the primers listed in Table 1 and inserted into the yeast expression vector pYES2 and transformed into the wild-type yeast strain BY4741. The transformed yeasts were selected on a SD medium without uracil (SD-Ura). Positive clones were cultured in SD-Ura liquid media with 2% glucose for growth assays, and 6 μl drops (diluted to an OD600 of 0.5) and three serial 1:10 dilutions were spotted on SD-Ura plates containing 0 or 30 μM CdCl_2_ in the presence of 2% galactose. The yeast was grown on the plates at 30°C for 3 d for the comparison.

### Statistical analysis

Data were analyzed by One-Way ANOVA using IBM SPSS statistics 19.0 (IBM SPSS Inc., Chicago, IL). Duncan’s test was used to determine the significant differences between means (p<0.05). A Michaelis-Menten model combined with a linear component was applied to mathematically resolve the concentration-dependent kinetics of Cd using SigmaPlot 12.0 (Systat Software Inc., Chicago, IL).

## Results

### Plant growth and leaf chlorophyll content in response to Fe deficiency

**Fig 1:**
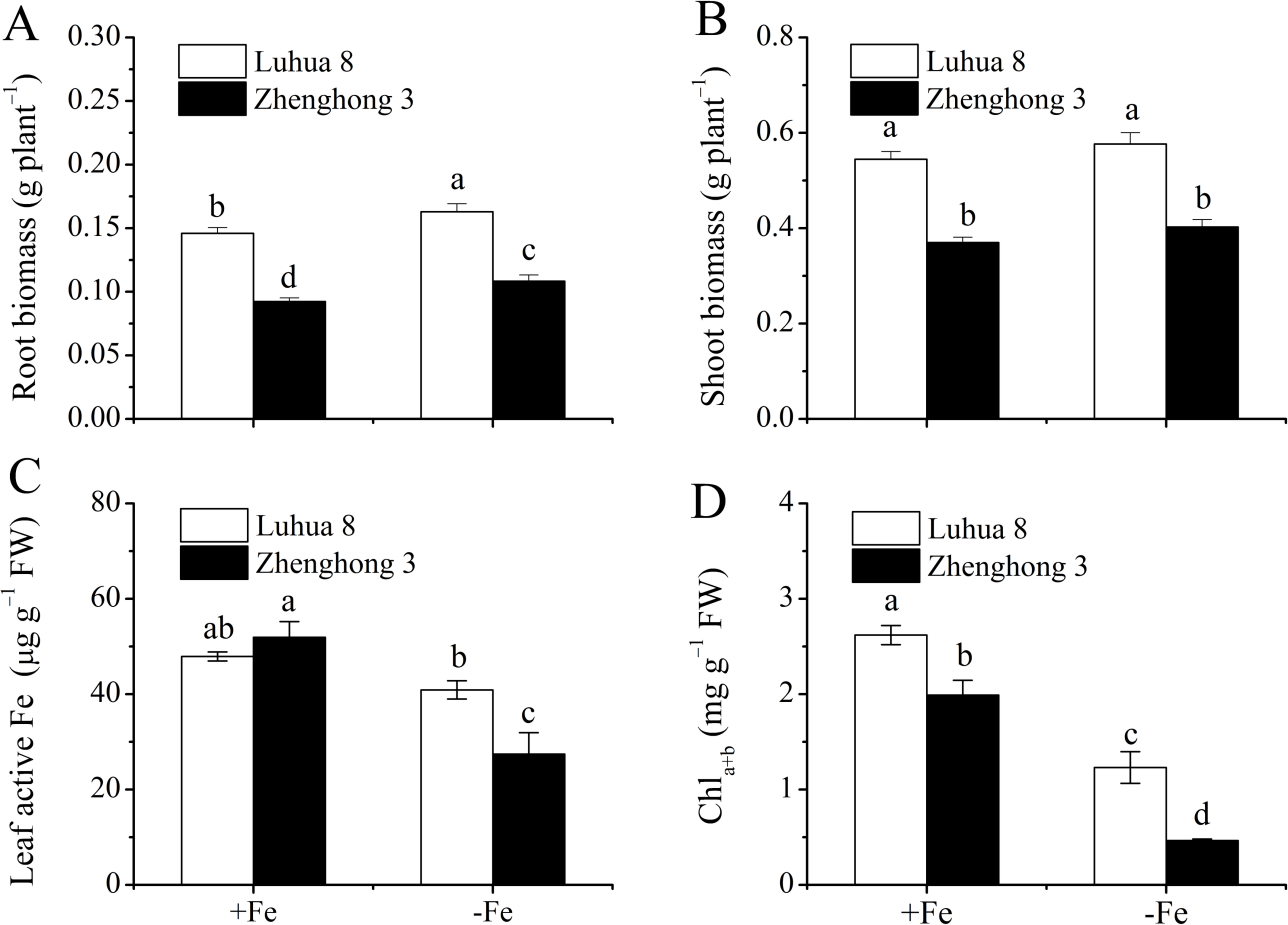
The biomasses of roots (a) and shoots (b), leaf active Fe contents (c) and chlorophyll contents (d) of Luhua 8 and Zhenghong 3 exposed to 0.2 μM CdCl2 for 12 d, under Fe-sufficient (+Fe) and -deficient (−Fe) conditions. Different letters above error bars indicate values (mean ± SE, n = 3) are significantly different between treatments at the 0.05 level.

The biomasses of the roots and shoots and leaf chlorophyll content in Luhua 8 were larger than that in Zhenghong 3 under both the Fe-sufficient and -deficient conditions (Fig 1A and 1B). Fe deficiency significantly enhanced the root biomass for both cultivars (Fig 1A), while the shoot biomasses were not affected (Fig 1B).

Active Fe contents in leaves of Fe-sufficient plants were similar between the two cultivars (Fig 1C). Fe-deficient treatment decreased the active Fe content in the leaves for both cultivars, and the decrease was more pronounced in Zhenghong 3 (by 47%) than in Luhua 8 (by 15%) (Fig 1C). In the case of the chlorophyll contents (Chl_*a+b*_), it was consistently higher in Luhua 8 than in Zhenghong 3. Fe-deficient treatment decreased the Chl_*a+b*_ by 53% and 77% for Luhua 8 and Zhenghong 3 respectively (Fig 1D).

### Influence of Fe deficiency on Cd accumulations in plants

**Fig 2:**
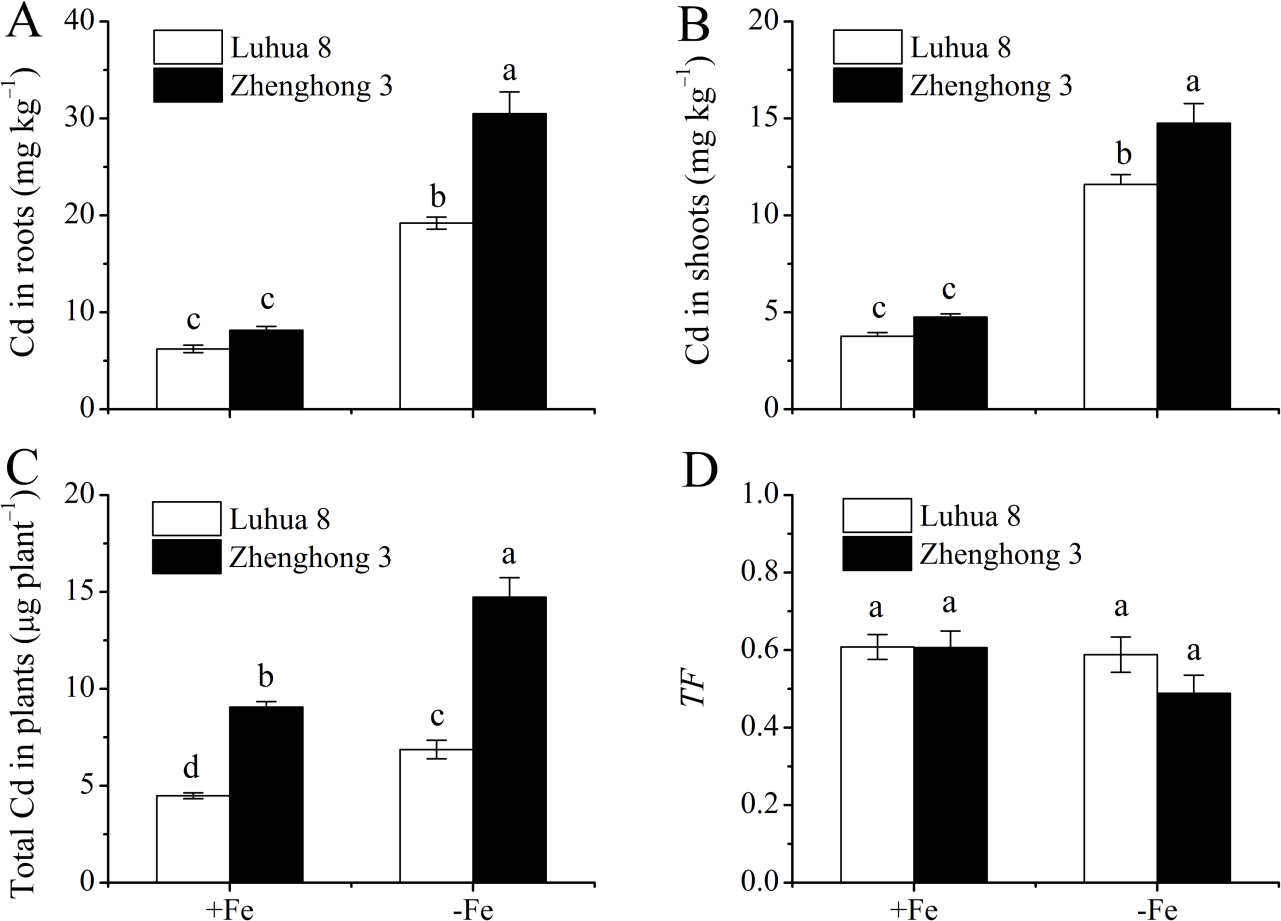
Cd concentrations in roots (a) and shoots (b), total Cd in plants (c) and TFs (d) in Luhua 8 and Zhenghong 3 exposed to 0.2 μM CdCl_2_ for 12 d, under Fe-sufficient (+Fe) and -deficient (−Fe) conditions. Different letters above error bars indicate values (mean ± SE, n = 3) are significantly different between treatments at the 0.05 level.

Accumulations of Cd in plants of the two cultivars followed an opposite pattern in comparison with Fe. Although Cd concentrations in the roots (Fig 2A) and shoots (Fig 2B) of Luhua 8, the low-Cd cultivar, were slightly lower than in those of Zhenghong 3, the high-Cd cultivar, the total Cd in plants was one fold higher in Zhenghong 3 than in Luhua 8 under Fe sufficient conditions (Fig 2C). Fe deficiency significantly increased Cd concentrations in the shoots and roots as well as the total Cd in plants of the two cultivars (Fig 2A–2C). The increases of Cd concentration in roots (Fig 2A) and total Cd in plants (Fig 2C) induced by Fe deficiency were higher in Zhenghong 3 (2.74- and 0.63-fold) than those in Luhua 8 (2.08- and 0.53-fold), whereas the increases of Cd concentration in shoots were similar between Luhua 8 (2.07-fold) and Zhenghong 3 (2.10-fold) (Fig 2B). The translocation factors of Cd from roots to shoots (TFs) were not significantly affected by cultivar and Fe treatments (Fig 2D).

### Concentration-dependent kinetics of Cd uptake in roots

**Fig 3:**
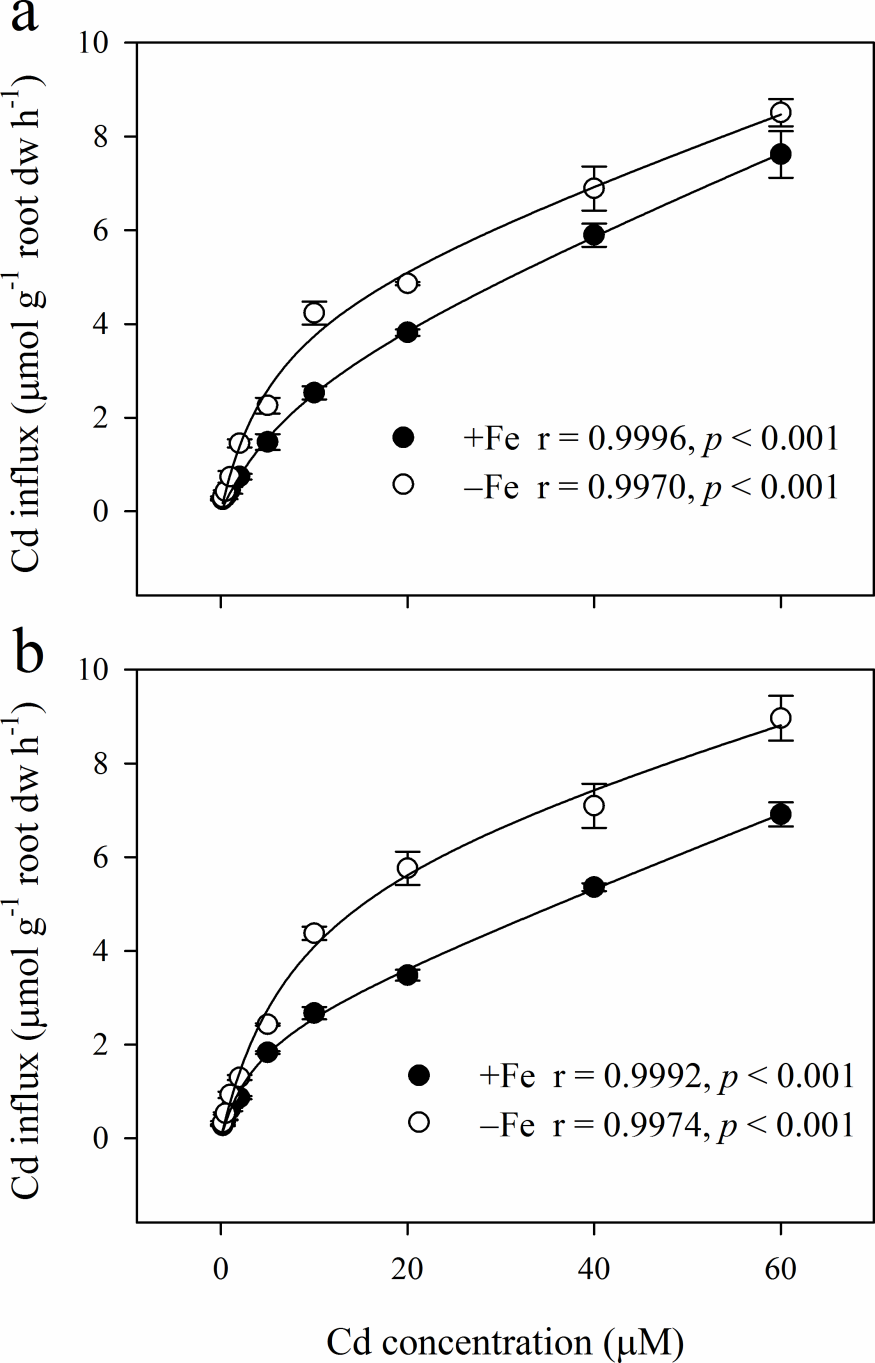
Concentration-dependent kinetics of Cd uptake in roots of Luhua 8 (a) and Zhenghong 3 (b) grown in full nutrient solution (+Fe) or without Fe (−Fe) for 12 d. Data points represent mean values ± SE, n = 3. The lines represent the best fit of the data using a Michaelis-Menten plus linear model.

The concentration-dependent kinetics of Cd influx showed a saturable (hyperbolic) component and a linear component for both cultivars (Fig 3A and 3B). In all cases, the model fitted closely the experimental data as demonstrated by R values of between 0.9970 and 0.9996 (Fig 3A and 3B).

Plants grown for 12 d in hydroponics without Fe showed obvious symptoms of chlorosis, indicating Fe deficiency; however, no decrease in the biomass of roots or shoots was observed (Fig 1). The Cd influx was similar between the two cultivars in Fe-sufficient conditions (Fig 3A and 3B). Fe deficiency considerably enhanced the rate of Cd influx in both cultivars, showing a cultivar-dependent relationship. A 1.56-fold increase in the maximal Cd influx (*V*_max_) was observed for Zhenghong 3 when the plants were Fe deficient compared with the treatment where Fe was supplied (Table 2). In the case of Luhua 8, only a 0.48-fold increase in *V*_max_ was induced by Fe deficiency. The *V*_max_ for Cd was similar between the two cultivars when the plants were grown in the presence of Fe. However, Zhenghong 3 showed a larger *V*_max_ than Luhua 8 under the conditions of Fe deficiency. The saturable component of the Cd influx was characterized by similar *K*_m_ values and significant differences were observed between cultivars and between Fe treatments. Compared with Luhua 8, Zhenghong 3 showed a higher *K*_m_ under Fe-sufficient conditions. Fe deficiency affected the *K*_m_ values in a cultivar dependent manner. Fe deficiency considerably enhanced the *K*_m_ in Zhenghong 3, whereas that in Luhua 8 was decreased. The angular coefficients characterizing the linear component of the Cd influx curves were slightly higher in Luhua 8 than in Zhenghong 3. Fe deficiency slightly but not significantly decreased the angular coefficients for both cultivars (Table 2).

**Table 2:** Parameters of the Michaelis-Menten plus linear model used to resolve the kinetic of influx curves in Fig 3. Data represent mean values ± SE.

Treatments	*V*_max_ (μmol g^−1^ root dw h^−1^)	*K*_m_ (μM)	Angular coefficient (μmol g^−1^ root dw h^−1^ μM^−1^)
Luhua 8			
+Fe	3.23±0.48	9.01±2.12	0.081±0.006
–Fe	4.78±0.90	5.64±2.03	0.068±0.014
Zhenghong 3			
+Fe	2.44±0.26	3.67±0.87	0.077±0.005
–Fe	6.25±1.19	7.62±2.46	0.055±0.017

### Expression of *AhIRT1* and *AhNRAMP1* in response to Fe deficiency

**Fig 4:**
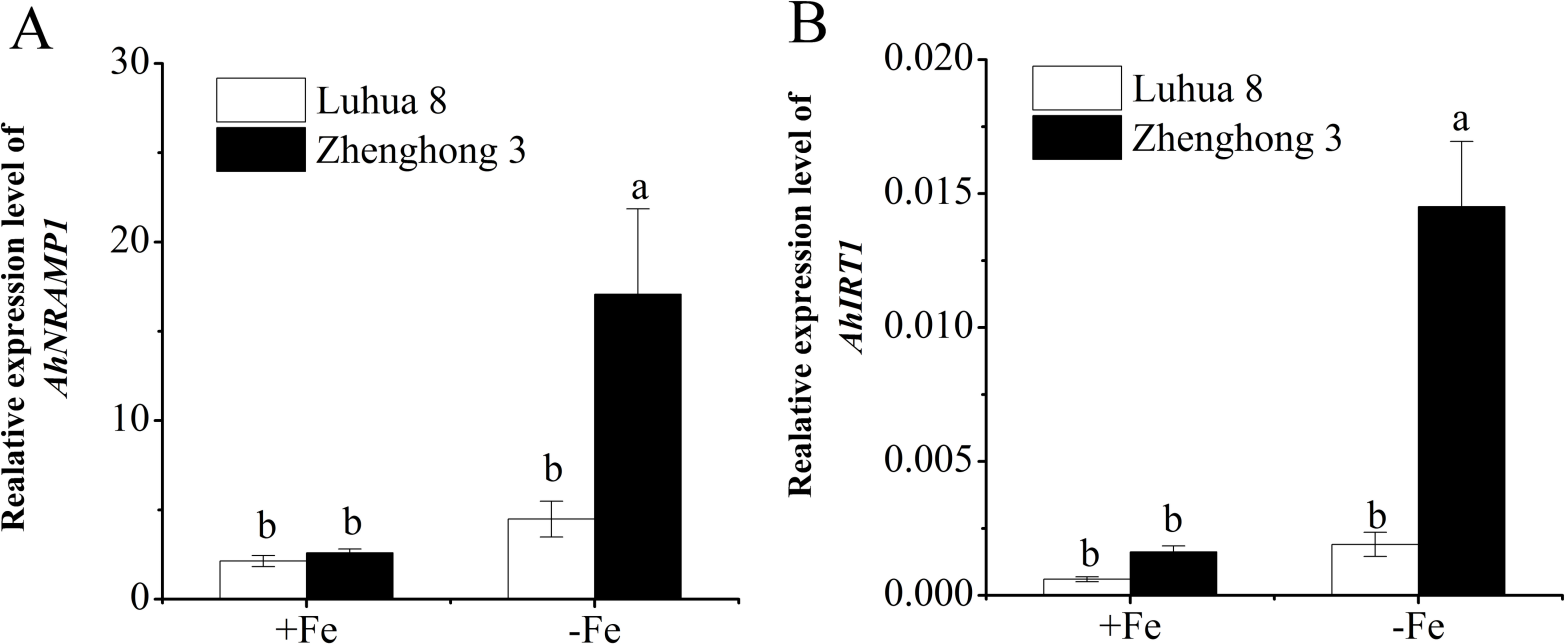
Expression pattern of *AhNRAMP1* (a) and *AhIRT1* (b) in the roots of Luhua 8 and Zhenghong 3 grown in full nutrient solution (+Fe) or without Fe (−Fe) for 12 d. The expression levels of *AhNRAMP1* and *AhIRT1* were normalized to that of *Ahactin* gene. Different letters above error bars indicate values (mean ± SE, n = 3) are significantly different between treatments at the 0.05 level.

Fig 4 shows the expression of *AhIRT1* and *AhNRAMP1* in the roots of Luhua 8 and Zhenghong 3 under Fe-sufficient and -deficient conditions. The two cultivars showed a similar expression of *AhNRAMP1* in the roots, while the expression of *AhIRT1* was slightly higher in Zhenghong 3 than in Luhua 8. Fe deficiency induced expression of *AhNRAMP1* and *AhIRT1* in the roots by 2.1- and 2.9-fold for Luhua 8, and by 6.6- and 9.0-fold for Zhenghong 3 respectively (Fig 4).

### Expression of AhIRT1 and AhNRAMP1 in yeast

**Fig 5:**
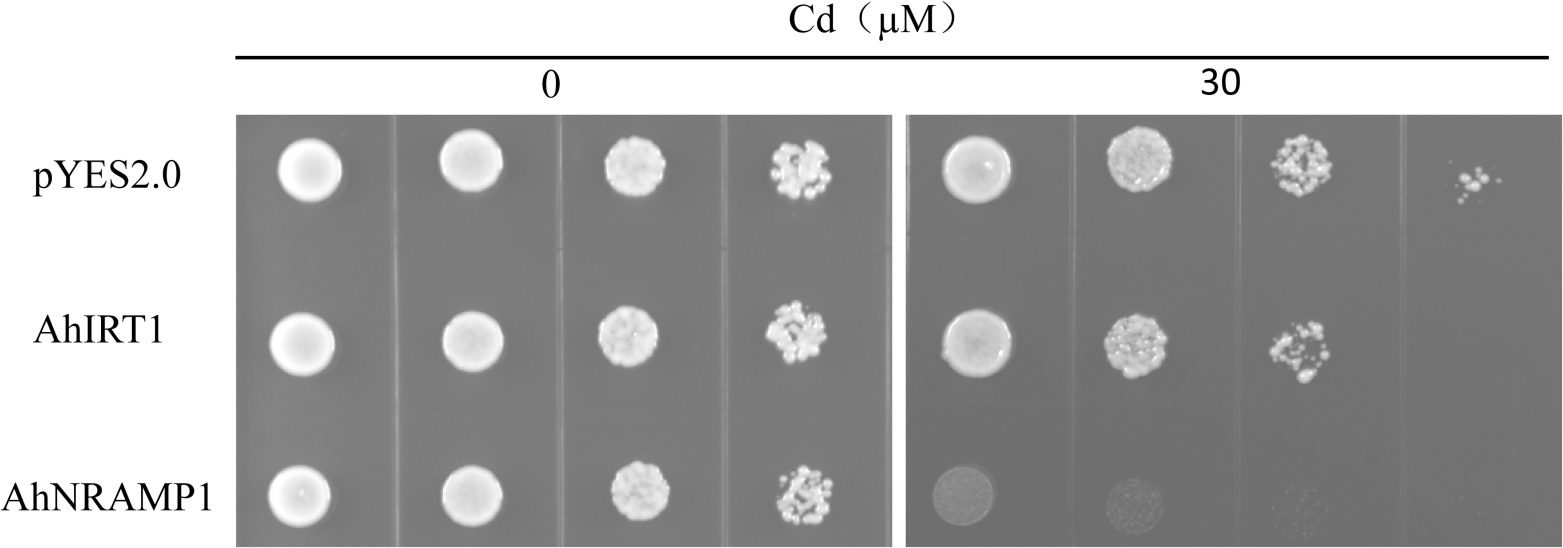
Growth of wild-type yeast cells transformed with empty vector pYES2, *AhIRT1* and *AhNRAMP1* in the presence of galactose. Serial dilutions of the transformed yeast cells with OD_600nm_ 0.5 to 0.0005 were spotted on SD-Ura plates containing 0 or 30 μM CdCl_2_ in the presence of galactose. The yeast was grown on the plates at 30°C for 3 d for the comparison.

To investigate whether AhIRT1 and AhNRAMP1 transport Cd, we carried out a yeast functional complementation assay using the wild type strain BY4741. In the presence of galactose, the yeast strains expressing *AhIRT1* or *AhNRAMP1* showed more sensitivity to Cd than the vector control (Fig 5), suggesting that AhIRT1 and AhNRAMP1 may function as transporters involved in Cd uptake.

### Root morphology of the two peanut cultivars in response to Fe deficiency

**Fig 6:**
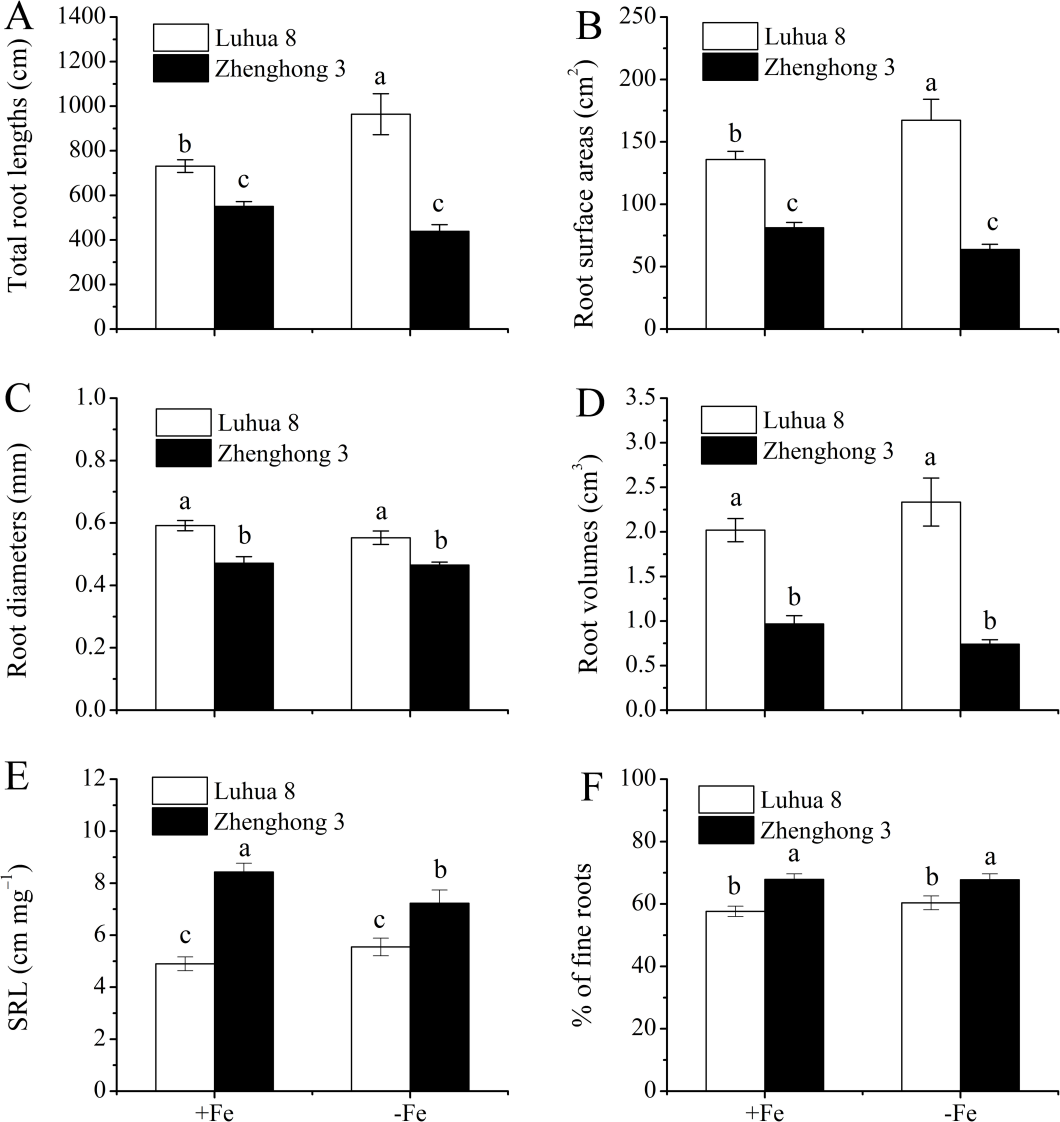
Root morphological traits of Luhua 8 and Zhenghong 3 grown in full nutrient solution (+Fe) or without Fe (−Fe) for 12 d. Different letters above error bars indicate values (mean ± SE, n = 3) are significantly different between treatments at the 0.05 level.

The two cultivars differed in root morphology in terms of RL, SA, RD and RV, and these parameters were generally higher in Luhua 8 than in Zhenghong 3 (Fig 6A–6D). Fe deficiency significantly increased the RL and SA in Luhua 8, while those in Zhenghong 3 remained unaffected (Fig 6A and 6B). The RD and RV in both cultivars were not affected by Fe deficiency.

The specific root length (SRL) was consistently higher in Zhenghong 3 than in Luhua 8. Fe deficiency caused a slight but not significant increase in the SRL of Luhua 8, while those in Zhenghong 3 significantly decreased (Fig 6E). In the case of the proportion of fine roots (0–0.4 mm diameter classes), it was consistently higher in Zhenghong 3 than in Luhua 8, and was not affected by Fe treatments (Fig 6F).

## Discussion

In previous papers, we have found that Luhua 8 and Zhenghong 3 differ from each other in Cd accumulation in both the seeds of mature plants and the shoots of seedlings [5, 25, 27, 28]. Differential responses in Cd accumulation to Fe deficiency were also observed between the two cultivars [25, 27]. The present results showed that, although the two cultivars were similar in the responses of plant growth to Fe deficiency (Fig 1A and 1B), the decreases in the active Fe content and chlorophyll contents in the leaves as a consequence of Fe deficiency were larger in Zhenghong 3 than in Luhua 8 (Fig 1C and 1D), indicating Zhenghong 3 is more sensitive to Fe deficiency compared with Luhua 8.

The results of the long-term accumulation experiment indicated that Zhenghong 3, the high seed-Cd cultivar, shows a high capacity for Cd uptake and accumulation in plants than Luhua 8, the low seed-Cd cultivar (Fig 2A–2C). The observation was in agreement with our previous results [5, 25, 27, 28]. Fe deficiency greatly enhanced Cd accumulation in plants for both cultivars (Fig 2A–2C), while the *TF*s were not affected (Fig 2D). The increases of Cd concentration in the roots and shoots were larger in Zhenghong 3 than in Luhua 8. The results indicate that increased Cd uptake by roots induced by Fe deficiency may account for higher Cd accumulation observed in Zhenghong 3. The increases in Cd accumulation as a consequence of Fe deficiency have been reported by other authors in various plants including peanut [6, 12, 25, 27, 31, 32].

The curves of the concentration-dependent kinetics of Cd influx were characterized by a saturable and a linear component in both cultivars (Fig 3). Similar results have been reported in various plants including wheat (*Triticum aestivum*) [33, 34], alpine pennycress (*Thlaspi caerulescens*) [35–37], maize [36, 38], pea (*Pisum sativum*) [6], *Arabidopsis halleri* [39] and *Sedum alfredii* [40]. According to previous studies [6, 31], the saturable component is generally considered as the symplastic absorption of Cd across the plasma membrane. Although the two cultivars showed a similar *V*_max_ of Cd influx under Fe-sufficient conditions, the *K*_m_ values were much lower in Zhenghong 3 than in Luhua 8 (Table 2). These results suggest that the higher Cd accumulation in Zhenghong 3 is a direct consequence of a higher affinity for Cd influx.

The two cultivars exhibited different responses of *V*_max_ and *K*_m_ values of Cd influx to Fe deficiency. In Zhenghong 3, Fe deficiency markedly increased both the *V*_max_ and *K*_m_ values of Cd influx. In Luhua 8, however, Fe deficiency slightly increased the *V*_max_ but greatly decreased the *K*_m_ (Table 2). These results indicate that a high *V*_max_ is probably more important than a low *K*_m_ for the increase of Cd influx induced by Fe deficiency in Zhenghong 3, while in Luhua 8, a higher affinity (lower *K*_m_) may be involved. The increase of the *V*_max_ for Cd influx induced by Fe deficiency in peanut seedlings were 0.48- and 1.56-fold for Luhua 8 and Zhenghong 3 respectively, the values were lower than that in pea (6.94-fold) [6] and *T*. *caerulescens* (9-fold) [31].

The angular coefficients characterizing the linear component of the influx curves that is generally considered to reflect Cd that remain bound to cell walls after desorption [6, 34, 37, 38, 40, 41]. In the present study, we found that the angular coefficients were similar between the two cultivars and shows a slight but nonsignificant reduction in response to Fe deficiency in both cultivars (Table 2). The results, consistent with the previous findings [6, 31], indicate that Fe deficiency had relatively little effect on the adsorption of Cd in the cell walls.

The root system is the main organ through which crops absorb water and mineral elements. Previous studies have demonstrated that Fe deficiency can alter the root morphological characteristics [2, 16], and the alterations were also proven to closely relate to the uptake and accumulation of Cd in plants [5, 17–19]. To examine the hypothesis that the changes in root morphology induced by Fe deficiency may be related to Cd uptake and accumulation in plants, root morphological responses to Fe deficiency were evaluated. The results obtained from the present study do not support the abovementioned hypothesis. For instance, although Fe deficiency caused greater increases in the *V*_max_ for Cd influx (Table 2) and Cd accumulation in the roots and shoots (Fig 2) in Zhenghong 3, the root morphological characteristics of this cultivar remained unaffected (Fig 6).

Additionally, we found that, although Luhua 8 shows higher RL, SA, RD and RV than Zhenghong 3 under Fe-sufficient conditions, the SRL and proportion of fine roots were greatly higher in Zhenghong 3 than in Luhua 8 (Fig 6E and 6F). The SRL represents the trade-offs between producing longer and thinner roots for resource acquisition (benefit) and partitioning more biomass for construction and maintenance (cost) [42], and it was shown to positively correlate with the uptake and accumulation of Cd in peanuts [5]. The fine roots have been demonstrated to play an important role in Cd uptake and translocation [5, 43–45]. Therefore, higher Cd accumulation in Fe-sufficient plants of Zhenghong 3 may, at least partially, result from their higher SRL and proportion of fine roots.

The molecular study provides a possible explanation to the physiological data presented. Compared with Luhua 8, Zhenghong 3 showed a slightly higher expression of *AhIRT1* and *AhNRAMP1* in the roots (Fig 4). The ability of IRT [6–8, 12, 31] and NRAMP [9–11] to transport Cd has been well established. In the present study, the heterologous assay in yeast also indicated that AhIRT1 and AhNRAMP1 may function as transporters of Cd in peanut (Fig 5). Thus, the initial expression of *AhIRT1* and *AhNRAMP1* in the roots may be responsible for high capacity for Cd uptake and accumulation in Zhenghong 3.

Fe deficiency increased the transcript abundance of *AhIRT1* and *AhNRAMP1* in the roots for both cultivars (Fig 4). Similar results have been reported in several plants [6, 8, 10, 12, 31, 46]. Induction of *AhIRT1* and *AhNRAMP1* by Fe deficiency was greater in Zhenghong 3 than in Luhua 8 (Fig 4). The findings correspond to the greatly increased *V*_max_ for Cd influx (Table 2) and Cd accumulation in roots and shoots (Fig 2). The lower induction of *AhIRT1* and *AhNRAMP1* is also consistent with the small increase in the *V*_max_ for Cd influx in Luhua 8. These results suggested that the higher expression of *AhIRT1* and *AhNRAMP1* may be involved in Cd uptake and accumulation in the two peanut cultivars in response to Fe deficiency.

## Conclusions

This study has clearly established that, compared with Luhua 8, Zhenghong 3 shows lower *K*_m_, higher SRL and proportion of fine roots, and slightly higher expression of *AhIRT1* and *AhNRAMP1* in the roots. These traits may be responsible for high capacity for Cd accumulation in Zhenghong 3. Fe deficiency induces considerable increase in the uptake and accumulation of Cd in plants for both cultivars. The increase of Cd accumulation as a consequence of Fe deficiency was greatly larger in Zhenghong 3 than in Luhua 8, in which a greater increase of *V*_max_ for Cd influx and higher expression of *AhIRT1* and *AhNRAMP1* are involved.

## Supporting information

S1 Data The data for Fig 1 in the manuscript. (OPJ)

S2 Data The data for Fig 2 in the manuscript. (OPJ)

S3 Data The data for Fig 3 in the manuscript. (JNB)

S4 Data The data for Fig 4 in the manuscript. (OPJ)

S5 Data The data for Fig 6 in the manuscript. (OPJ)
